# HY5: A Pivotal Regulator of Light-Dependent Development in Higher Plants

**DOI:** 10.3389/fpls.2021.800989

**Published:** 2022-01-17

**Authors:** Yuntao Xiao, Li Chu, Yumeng Zhang, Yeting Bian, Jiahui Xiao, Dongqing Xu

**Affiliations:** State Key Laboratory of Crop Genetics and Germplasm Enhancement, National Center for Soybean Improvement, College of Agriculture, Nanjing Agricultural University, Nanjing, China

**Keywords:** HY5, photomorphogenesis, root growth, nutrient utilization, pigment accumulation

## Abstract

ELONGATED HYPOCOTYL5 (HY5), a bZIP-type transcription factor, acts as a master regulator that regulates various physiological and biological processes in plants such as photomorphogenesis, root growth, flavonoid biosynthesis and accumulation, nutrient acquisition, and response to abiotic stresses. HY5 is evolutionally conserved in function among various plant species. HY5 acts as a master regulator of light-mediated transcriptional regulatory hub that directly or indirectly controls the transcription of approximately one-third of genes at the whole genome level. The transcription, protein abundance, and activity of HY5 are tightly modulated by a variety of factors through distinct regulatory mechanisms. This review primarily summarizes recent advances on HY5-mediated molecular and physiological processes and regulatory mechanisms on HY5 in the model plant *Arabidopsis* as well as in crops.

## Introduction

Plants utilize light as the predominant energy source for photosynthesis. Besides, light signal acts as an essential external factor that mediates a variety of physiological and developmental processes in plants (Paik and Huq, 2019; Song et al., 2020a; Xu, 2020). Plants are continuously exposed to dynamically changing light signals due to the daily and seasonal alternation in natural conditions. The various light signals are perceived by at least five classes of wavelength-specific photoreceptors including phytochromes (phyA-phyE), cryptochromes (CRY1 and CRY2), phototropins (PHOT1 and PHOT2), F-box containing flavin binding proteins (ZTL, FKF1, and LKP2), and UV-B RESISTANCE LOCUS 8 (UVR8; Paik and Huq, 2019). These photoreceptors are biologically activated by various light signals, subsequently initiating a large scale of transcriptional reprogramming at the whole genome level (Jing and Lin, 2020). Extensive genetic and biochemical studies have established that the ELONGATED HYPOCOTYL5 (HY5), a bZIP-type transcription factor, tightly controls the light-regulated transcriptional alternation. Loss of HY5 function mutant seedlings displays drastically elongated hypocotyls in various light conditions (Oyama et al., 1997), suggesting that HY5 acts downstream of multiple photoreceptors in promoting photomorphogenesis in plants. In addition to inhibiting hypocotyl growth, HY5 regulates other various physiological and developmental processes including root growth, pigment biosynthesis and accumulation, responses to various hormonal signals, and low and high temperatures (Nawkar et al., 2017; van Gelderen et al., 2018; Zhang Y. et al., 2019; Li J. et al., 2020; Marzi et al., 2020; Ortigosa et al., 2020; Yadukrishnan et al., 2020; Bhagat et al., 2021; Wang et al., 2021a,b). This review summarizes the recent advances and progresses on HY5-regulated cellular, physiological, and developmental processes in various plant species. We also highlighted emerging insights regarding the HY5-mediated integration of multiple developmental, external, and internal signaling inputs in the regulation of plant growth.

## HY5 is Evolutionally Conserved in Plant Species

**FIGURE 1:**
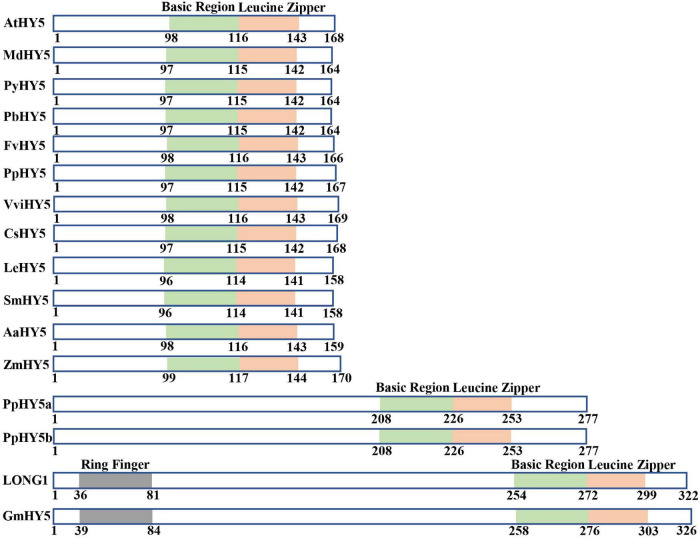
HY5 orthologs are evolutionally conserved in protein structure. Schematic representations of domains present in multiple HY5 orthologs. AtHY5 (Oyama et al., 1997), MdHY5 (An et al., 2017a), PyHY5 (Wang et al., 2020), PbHY5 (Wu et al., 2019), FvHY5 (Li Y. et al., 2020), PpHY5 (Zhao et al., 2017), VviHY5 (Loyola et al., 2016), CsHY5 (Huang et al., 2019), LeHY5 (Liu et al., 2004), SmHY5 (Jiang et al., 2016), AaHY5 (Hao et al., 2019), ZmHY5 (Huai et al., 2020), PpHY5a and PpHY5b (Yamawaki et al., 2011), LONG 1 (Weller et al., 2009), and GmHY5 (Song et al., 2008). Numbers indicate the position of amino acid residues.

**FIGURE 2:**
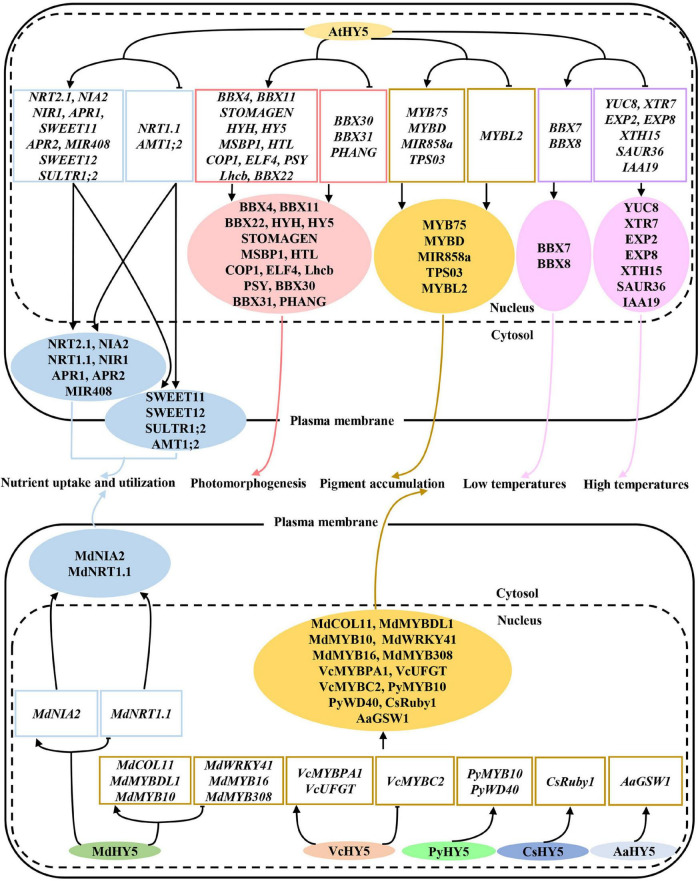
HY5 regulates a variety of physiological and developmental processes in diverse plant species. Various HY5 orthologs are involved in the control of photomorphogenesis, nutrient utilization, pigment accumulation, and low and high temperature signaling by controlling downstream target genes.

HY5 is originally identified as a positive regulator of photomorphogenesis, root gravitropic response, and lateral root development in the model plant *Arabidopsis* (Oyama et al., 1997). *HY5* gene encodes a bZIP-type transcription factor that controls approximately one-third of the expression of genes throughout the whole genome (Lee et al., 2007; Burko et al., 2020b). Extensive studies have revealed that HY5 regulates a variety of developmental processes, responsiveness of various hormonal and environmental signals through divergent but overlapping signaling networks in plants (Gangappa and Botto, 2016; Su et al., 2021). The orthologs of HY5 in distinct plant species are highly conserved in protein structure and function (Figure 1). HY5 from most plant species possess a basic region and a Leucine Zipper Domain responsible for DNA binding and dimerization, respectively, and the others contain an additional RING-finger motif in some plant species such as soybean and pea (Figure 1). These imply HY5 orthologs likely exert common but divergent functions in regulating physiological and developmental processes among various plant species. The HY5 orthologs in various plant species have been shown to mediate multiple light-regulated development and response. The HY5 in *Arabidopsis*, soybean, pea, apple, moss, tomato, rice, and maize regulate the hypocotyl or stem growth, shade avoidance, and responses to internal signals (e.g., GA and auxin) and external signals (e.g., light, low, and high temperatures) (Oyama et al., 1997; Yamawaki et al., 2011; An et al., 2017b; Burman et al., 2018; Wang et al., 2018; Huai et al., 2020; Lyu et al., 2021). Sweet wormwood, sweet orange, strawberry, pear, peach, tomato, eggplant, and grape HY5 orthologs are involved in the regulation of light-induced flavonoid biosynthesis and accumulation (Loyola et al., 2016; Li J. et al., 2017; Liu et al., 2018; Hao et al., 2019; Huang et al., 2019; Wu et al., 2019; Li Y. et al., 2020; Wang et al., 2020; Zhao et al., 2021; Figure 2). The biochemical functions of HY5 are conserved in distinct plant species. HY5 acts as a transcription factor that predominantly binds to the *ACGT*-containing *cis*-element (e.g., *G-box* and *T/G-box*) and controls the expression of numerous target genes in response to light signals, which in turn serves to modulate distinct light-regulated physiological and developmental processes in plants.

## HY5 is a Central Regulator of Light Signaling

Light tightly controls the seedling development including inhibition of hypocotyl growth, promotion of cotyledon expansion, and accumulation of chlorophyll which is totally termed photomorphogenesis. HY5 acts as an essential and indispensable regulator of this developmental process in *Arabidopsis*. More importantly, its abundance is correlated with seedling photomorphogenesis. A variety of factors converge on HY5, which indirectly or directly controls the expression of over 3,000 genes, to ensure normal seedling photomorphogenic development in response to dynamically changing light signals.

In the darkness, CONSTITUTIVELY PHOTOMORPHOGENIC 1 (COP1)-SUPPRESSOR OF PHYTOCHROME A-105 (COP1-SPA) E3 ligase complex directly targets HY5 for polyubiquitination and degradation, and thus, the abundance of HY5 remains at an extremely low level (Osterlund et al., 2000; Han et al., 2020). COP1 SUPPRESSOR 1 (CSU1), CSU2, PHYTOCHROME INTERACTING 1 (PIF1), and SIZ1 act as negative regulators of COP1-SPA complex to ultimately maintain HY5 homeostasis in etiolated seedlings (Xu D. et al., 2014; Xu X. et al., 2014; Xu et al., 2015; Lin et al., 2016). On light irradiation, the activity of COP1-SPA is largely suppressed through multiple regulatory mechanisms. The photoreceptors phyA, phyB, CRY1, and CRY2 directly associate with COP1-SPA to disrupt the formation of the COP1-SPA complex (Podolec and Ulm, 2018). Meanwhile, light-activated CRY1, CRY2, and UVR8 compete with HY5 for COP1 binding through conserved Val-Pro (VP) motifs (Lau et al., 2019; Ponnu et al., 2019). As a long strategy, COP1 migrates from the nucleus to the cytoplasm under prolonged light illumination (Han et al., 2020; Ponnu and Hoecker, 2021). Together, all these molecular regulatory mechanisms consequently serve to trigger the appropriate accumulation of HY5 in the light. Accumulated HY5 directly or indirectly regulates the expression of over 3,000 genes, thereby controlling diverse physiological growth and responses to various hormonal and environmental signals (Lee et al., 2007; Burko et al., 2020b).

**FIGURE 3:**
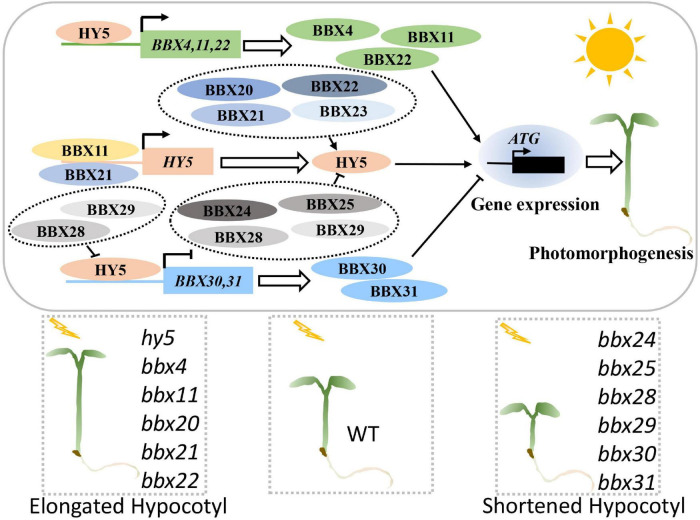
BBX-HY5 regulatory module promotes photomorphogenesis. BBXs and HY5 form a complex transcriptionally regulatory network in promoting photomorphogenesis. HY5 activates the expression of *BBX4*, *BBX11*, and *BBX22*. BBX11 and BBX21 positively control the transcription of *HY5*. BBX20, BBX21, BBX22, and BBX23 form heterodimers with HY5 to enhance its activity, whereas BBX24, BBX25, BBX28, and BBX29 interact with HY5 to inhibit its transcriptional activation activity. BBX28 and BBX29 inhibit the HY5 action to upregulate *BBX30* and *BBX31* at the transcriptional level. Together, these events synergistically serve to control HY5-mediated genes to promote photomorphogenesis. HY5, BBX4, BBX11, BBX20, BBX21, and BBX22 are positive regulators of light signaling, while BBX24, BBX25, BBX28, BBX29, BBX30, and BBX31 negatively regulate photomorphogenesis.

HY5 together with a group of B-box proteins (BBXs) work in concert to control the expression of numerous downstream target genes as well as multiple molecular and biological events (Figure 3). BBXs function as rate-limiting cofactors in mediating the molecular action of HY5. BBX20, BBX21, BBX22, and BBX23 enhance the transcriptional activation activity of HY5 by forming heterodimers (Zhang et al., 2017; Bursch et al., 2020), whereas BBX24, BBX25, BBX28, and BBX29 repress HY5 biochemical activity through a similar regulatory mechanism (Gangappa et al., 2013; Lin et al., 2018; Song et al., 2020b). In addition, BBX11 and BBX21 directly bind to the promoter regions of *HY5* to activate its transcription, while HY5 positively regulates the expression of *BBX11*, *BBX21*, and itself, thus forming a transcriptional feedback loop in controlling downstream target gene expression (Xu D. et al., 2016, 2018; Zhao et al., 2020; Job and Datta, 2021; Song et al., 2021). These findings suggest that a subgroup of BBXs and HY5 forms a complex transcriptional network that orchestrates the expression of light-responsive genes. Furthermore, HY5 positively controls *BBX4* and *BBX22*, while negatively controls *BBX30* and *BBX31* at the transcriptional level (Chang et al., 2008; Heng et al., 2019; Yadav et al., 2019; Liu et al., 2021), suggesting that HY5 also modulates the function of some BBXs by regulating their transcript levels in the light. BBX-HY5 regulatory module likely plays a critical role in the regulation of the expression of numerous light-responsive genes, through which HY5 controls diverse light-dependent development in plants.

In addition to BBXs, other components also mediate the light signal transduction by modulating HY5 activity and/or transcription. TEOSINTE BRANCHED1/CYCLOIDEA/PROLIFERATING CELL FACTOR (TCP2) and SHI-RELATED SEQUENCE 5 (SRS5) positively control the transcription of *HY5* to promote photomorphogenesis (He et al., 2016; Yuan et al., 2018). INOSITOL REQUIRING 80 (INO80) affects the chromatin modifications of *HY5* and represses its transcription to inhibit photomorphogenic development (Yang C. et al., 2020). COLD REGULATED 27 (COR27) and COR28 directly interact with HY5 to enhance its transcriptional activation activity toward downstream target genes, consequently leading to the promotion of photomorphogenic development (Li X. et al., 2020; Zhu et al., 2020). HY5 associates with HISTONE DEACETYLASE 15 (HDA15) and recruits it to the promoter regions of target genes, thereby decreasing the levels of histone H4 acetylation in a light-dependent manner and repressing their transcription (Zhao et al., 2019). Similarly, HY5 recruits the SWI-INDEPENDENT3 LIKE (SNL)-HDA19 deacetylase complex to the chromatin regions of *BBX22* and itself, which in turn, decreases the accessibility and histone acetylation and suppresses their transcription (Jing et al., 2021). MYC2, MYC3, and MYC5 bind to *E-box cis*-element present in the *HY5* promoter to activate its expression, while HY5 inhibits the expression of *MYC2*, suggesting that MYCs and HY5 likely form a negative feedback loop in the regulation of seedling development (Chakraborty et al., 2019; Yi et al., 2020). These results indicate that plants acquired a complicated but delicate regulatory mechanism to fine-tune the HY5 transcript level and activity in the control of photomorphogenesis.

HY5 directly binds to the *G-box cis*-element present in *TANDEM ZINC-FINGER/PLUS3* (*TZP*) promoter to activate its expression in the far-red light. In turn, TZP competes with COP1 for binding of HY5, thus leading to the accumulation of HY5 that promotes phyA signaling (Li C. et al., 2021). Blue light-activated CRY1 competes with AGB1 for binding of HY5, thus leading to the enhanced biochemical activity of HY5 in promoting photomorphogenesis (Lian et al., 2018). Meanwhile, CRY1 interacts with SWC6 and ARP6 and stabilizes HY5 in blue light. Stabilized HY5 recruits SWR1 complex to HY5 target loci to regulate the transcription of its target genes and photomorphogenesis (Mao et al., 2021b). In response to UV-B light signals, WRKY DNA-BINDING PROTEIN 36 (WRKY36) represses the *HY5* at the transcriptional level to inhibit photomorphogenesis (Yang et al., 2018). These results suggest that distinct light signals perceived by different wavelength-specific photoreceptors modulate the seedling growth at least in part through HY5 and HY5-mediated signaling.

## HY5 Controls Light-Mediated Root Growth

Although roots grow in the soil, light signaling transduced from shoot to root affects lateral and primary root development in plants (Yang and Liu, 2020). Light triggers the accumulation of HY5 that positively regulates root growth and development under soil-grown conditions (Lee et al., 2016b; Zhang Y. et al., 2019). HY1 activates the transcription of *HY5* in the root cells. Subsequently, HY5 promotes the accumulation of plant phytohormone auxin in the oscillation zone, leading to lateral growth and branching (Duan et al., 2021). Light-induced HY5 in the root cells activates the transcription of *LAZY4* to promote root gravitropism (Yang P. et al., 2020). Far-red, red, and blue light perceived by PHYs and CRYs in the shoot regulate lateral and primary root growth through the HY5 (Lee et al., 2016a; van Gelderen et al., 2018; Gao et al., 2021). HY5 is induced by far-red light in the lateral root primordia in a phytochrome-dependent manner. HY5 decreases the abundance of auxin transporters PIN-FORMED3 and LIKE-AUX1 3 in the plasma membrane to inhibit lateral growth under low red:far-red light conditions (van Gelderen et al., 2018). Red and blue lights stabilize the HY5 dependent on phyB or CRYs in the root, where it activates the *miR163* and itself to promote primary root growth (Gao et al., 2021; Li T. et al., 2021). Thus, these results suggest that distinct wavelength-specific photoreceptors transduce the light signals to HY5 in the root cells where it regulates the root growth in the soil.

## HY5 Regulates the Pigment Accumulation

Anthocyanins are a class of flavonoids that provide protection against biotic and abiotic stresses. HY5 integrates distinct environmental signals such as light, low and high temperatures, salinity, and drought stresses in the control of anthocyanin biosynthesis. HY5 not only directly activates the *MYB12* transcription but also directly binds to the promoter regions of multiple anthocyanin biosynthetic genes to activate their transcription (Shin et al., 2007; Bhatia et al., 2021). In addition, HY5 represses the expression of *MYB-LIKE 2 (MYBL2)*, which is a negative regulator of anthocyanin biosynthesis (Nguyen et al., 2015; Wang et al., 2016; Kim et al., 2017). Consequently, these molecular events contribute to anthocyanin biosynthesis and accumulation. In apple, MdHY5 interacts with MdBBX22 to promote the expression of genes involved in anthocyanin biosynthesis (Henry-Kirk et al., 2018; An et al., 2019). Meanwhile, MdHY5 directly activates *CONSTANS-LIKE 11* (*MdCOL11)*, *MdMYBDL1*, *MdMYB10*, and itself but represses *MdWRKY41* transcript level, which in turn promote anthocyanin biosynthesis (Bai et al., 2014; An et al., 2017a,^2020^; Liu et al., 2019; Mao et al., 2021a). In tomatoes, SlHY5 also controls the expression of anthocyanin biosynthetic genes to promote anthocyanin accumulation (Liu et al., 2018; Qiu et al., 2019). Red pear PyHY5 alone or together with PyBBX18 promotes the expression of *PyMYB10* and *WD40 PROTEIN GENE* (*PyWD40)*, leading to the anthocyanin biosynthesis (Bai et al., 2019; Wang et al., 2020). Moreover, the other HY5 orthologs in multiple plant species such as strawberry, blood orange, grape, peach, and eggplant have also been shown to promote the light-induced anthocyanin biosynthesis and accumulation (Li J. et al., 2017; Huang et al., 2019; Li Y. et al., 2020; Sun et al., 2020; Zhao et al., 2021). All these facts suggest that HY5 is an essential regulator of anthocyanin biosynthesis and accumulation, and its function in promoting this physiological process is conserved among diverse plant species.

Besides, light-induced HY5 is also involved in many other secondary metabolite biosynthesis and accumulation in plants. Knock-down *LeHY5* transcription leads to reduced carotenoid levels and pale green immature fruits and leaves, indicating that LeHY5 promotes the carotenoid-mediated fruit ripening in tomatoes (Liu et al., 2004). Consistently, LeHY5 regulates the transcription of genes involved in carotenoid, anthocyanin biosynthesis, and ethylene signaling (Wang et al., 2021d). The expression level of *PaHY5* in apricot fruit is correlated with the content of carotenoids during the ripening process, indicating that PaHY5 positively controls carotenoid biosynthesis and accumulation in apricot fruit (Zhang L. et al., 2019). In *Artemisia annua*, AaHY5 activates *GLANDULAR TRICHOME-SPECIFIC WRKY1* (*AaGSW1*) and *AaWRKY9* in a light-dependent manner to promote artemisinin biosynthesis (Hao et al., 2019; Fu et al., 2021). HY5 upregulates the expression of *TERPENE SYNTHASE 03* (*TPS03)*, a terpene biosynthetic gene, to facilitate the terpenoid biosynthesis in *Arabidopsis* (Michael et al., 2020). In summary, all these studies suggest that light-controlled HY5 plays a critical role in the regulation of multiple secondary metabolite biosynthesis in different plant species.

## HY5 Functions in the Regulation of Nutrient Uptake and Utilization

Nutrient acquisition and utilization is essential and necessary for plant growth and development. HY5 controls the expression of a set of genes involved in nitrogen uptake and transport including *NITRATE TRANSPORTER 1.1 (NRT1.1)*, *NITRATE TRANSPORTER 2.1* (*NRT2.1), AMMONIUM TRANSPORTER 1*, *2* (*AMT1;2)*, *NITRATE REDUCTASE 2* (*NIA2)*, and *NITRITE REDUCTASE 1* (*NIR1*; Jonassen et al., 2009; Huang et al., 2015; Chen et al., 2016; Sakuraba and Yanagisawa, 2018). HY5 also activates the expression of two sucrose efflux genes *SUCROSE TRANSPORTER 11 (SWEET11)* and *SUCROSE TRANSPORTER 12 (SWEET12)* by directly associating with their promoters (Chen et al., 2016; Sakuraba and Yanagisawa, 2018). It has been shown that HY5 moves from shoot to root, where it promotes root growth and nitrate uptake. In the shoot, HY5 facilitates carbon assimilation and translocation, while it activates the nitrate transporter *NRT2.1* to enhance nitrate uptake and utilization in the root cells (Chen et al., 2016). A very recent study has shown that HY5 protein mobility is likely not required for shoot-to-root communication. A mobile signal acting downstream of HY5 may function in the shoot-to-root communication (Burko et al., 2020a). Red light activated phyB promotes the accumulation of HY5 both in the shoot and root. A portion of HY5 in the shoot moves to the root, together with root localized HY5, and directly regulates the phosphate starvation-responsive genes to facilitate phosphorus acquisition in *Arabidopsis* (Sakuraba et al., 2018). SQUAMOSA PROMOTER BINDING PROTEIN-LIKE7 (SPL7) and HY5 act coordinately to regulate the transcription of *MIR408* and its target genes, resulting in the alternation of copper allocation to the chloroplast and plastocyanin levels (Zhang et al., 2014).

**FIGURE 4:**
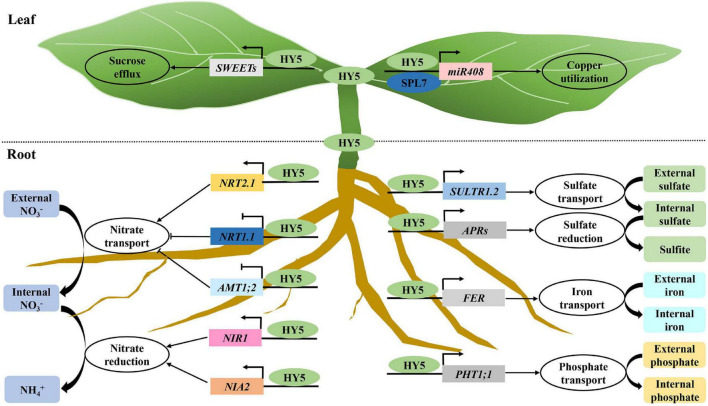
HY5 acts as a central hub of nutrient signaling. Light triggers the accumulation of HY5 both in the shoot and root. HY5 activates the transcription of *SWEETs* and *miR408* to promote sucrose and copper utilization. A portion of shoot localized HY5 proteins move to the root. HY5 proteins in the root regulate the transcription of genes involved in the uptake and/or transport of nitrogen, sulfur, Fe, and phosphate, thereby facilitating multiple nutrient assimilation.

In apple, MdHY5 promotes nitrate assimilation by positively regulating the expression of *MdNIA2* and *MdNRT1.1* (An et al., 2017a). In tomatoes, SIHY5 controls starch degradation and carbon utilization by directly associating with the promoter regions of starch degradation-related genes (e.g., *PWD*, *BAM1*, *BAM3*, *BAM8*, *MEX1*, and *DPE1*) to activate their transcription (Dong et al., 2021). SIphyB promotes light-induced Fe uptake in tomatoes by promoting the accumulation of SIHY5. SIHY5 moves from shoot to root, where it activates the expression of the *FER* transcription factor, leading to the increase of Fe uptake (Guo et al., 2021). Similarly, red light-activated SIphyB enhances the SIHY5 action in the shoot. Therefore, shoot SIHY5 moves to the root to promote phosphate uptake under phosphate starvation conditions (Ge et al., 2021). Altogether, these results suggest that HY5 is necessary and required for precisely controlling multiple nutrient uptake and utilization in diverse plant species in response to fluctuating light signals (Figure 4).

## HY5 Mediates the Responsiveness of Ambient Low and High Temperatures

As sessile organisms, plants have to cope with the fluctuating temperatures in adapting to daily and seasonal changing cycles. Low temperature is one of the most impactful environmental cues that affect plant growth and development. HY5 controls the expression of approximately 10% of all cold-induced genes to promote cold acclimation in *Arabidopsis*. Low temperatures trigger the COP1 translocate from the nucleus to the cytoplasm, thus leading to the inactivation of COP1 and accumulation of HY5 (Catalá et al., 2011). PREFOLDIN 4 (PFD4) accumulates in the nucleus, where it interacts with HY5 to facilitate its polyubiquitination and degradation in a COP1-independent manner in response to low temperatures (Perea-Resa et al., 2017). Low temperatures stabilize phosphorylated blue light photoreceptor CRY2 which competes with COP1 to interact with HY5, thereby allowing the accumulation of HY5 that activates the expression of *BBX7* and *BBX8*. In turn, BBX7 and BBX8 regulate the transcription of a set of cold-responsive genes to promote freezing tolerance in plants (Li Y. et al., 2021). In tomatoes, SIHY5, SIMYB15, and SICBFs work synergistically in response to cold (4°C). On the one hand, SIHY5 positively regulates *SIMYB15* transcription. On the other hand, both SIHY5 and SIMYB15 upregulate the transcript levels of *SICBF1*, *SICBF2*, and *SICBF3*. Thus, these molecular events increase the cold tolerance in tomatoes (Zhang et al., 2020). MdHY5 and MdMYB108L form a transcriptional feedback loop to promote cold tolerance both dependent and independent on CBF signaling in apple (An et al., 2017b; Wang et al., 2019). Thus, HY5 regulates the cold accumulation both independent of and dependent on CBF signaling in plants.

At elevated high temperatures, HY5 abundance is dramatically reduced due to the inhibition of COP1 activity, resulting in thermomorphogenic development (Kim et al., 2017; Park et al., 2017). In contrast, HY5 competes with PIF4 for repressing PIF4-regulated gene expression and thermomorphogenesis (Gangappa and Kumar, 2017). Shoot and root growth occur simultaneously during early seedling development at high ambient temperatures (Bellstaedt et al., 2019). HY5 is required for controlling root thermomorphogenesis (Gaillochet et al., 2020; Lee et al., 2021). SPA directly phosphorylates HY5 to control its stability, through which HY5 regulates a set of auxin and BR-mediated gene expression in the root cells, consequently promoting root thermomorphogenesis (Lee et al., 2021; Wang et al., 2021c). Therefore, high temperatures tightly control the mode of HY5 action that contributes to both shoot and root thermosensory growth in plants.

## Concluding Remarks and Future Perspectives

Numerous studies have established that HY5 plays pleiotropic roles in regulating various physiological and developmental processes and responses to diverse internal and external signals in plants. A group of components converges on HY5 to modulate its abundance, activity, and transcription in maintaining its appropriate biological action. HY5 acts as a signaling hub that controls the expression of a large number of genes in response to dynamic changing developmental, hormonal, and environmental signals. This mechanistic regulation may ensure the plants adapt to the intracellular and surrounding fluctuating cues throughout their entire life cycles. Increasing studies have shown that HY5 functions are evolutionally conserved among various plant species. The HY5 orthologs in crops control multiple agronomic traits such as stem growth, root growth, nutrient uptake, and fruit ripening. Fulfilling a comprehensive understanding of HY5 functions and signaling will provide novel knowledge and strategies for the improvement of specific agronomic traits in crops. According to current fundamental knowledge on HY5 function and HY5-mediated signaling network, HY5 most likely have positive roles in the control of various physiological and developmental processes. It is therefore loss of HY5 function in different plant species such as *Arabidopsis*, rice, and soybean, leading to drastically deficient in many facets of development and growth. Increasing HY5 abundance or activity may be a helpful strategy to improve specific agronomic traits in crops. On the one hand, genetic engineering techniques could be applied to generate specific plants expressing appropriately increased HY5 abundance. On the other hand, manipulation of positive or negative regulators of HY5 could be used to enhance the HY5 action. In view of the complexity of HY5 signaling, further studies are required to clarify the detailed HY5 signaling network in diverse plant species.

## Author Contributions

All authors listed have made a substantial, direct, and intellectual contribution to the work, and approved it for publication.

## Conflict of Interest

The authors declare that the research was conducted in the absence of any commercial or financial relationships that could be construed as a potential conflict of interest.

## Publisher’s Note

All claims expressed in this article are solely those of the authors and do not necessarily represent those of their affiliated organizations, or those of the publisher, the editors and the reviewers. Any product that may be evaluated in this article, or claim that may be made by its manufacturer, is not guaranteed or endorsed by the publisher.
